# Highly efficient preparation of 1-lysophosphatidylcholine via high proportion of Novozym® 435 (lipase B from *Candida antarctica*)-catalyzed ethanolysis

**DOI:** 10.1016/j.btre.2020.e00505

**Published:** 2020-07-22

**Authors:** Sayumi Yasuda, Yukihiro Yamamoto

**Affiliations:** Faculty of Bioresource Sciences, Prefectural University of Hiroshima, Nanatsuka-cho, 5562, Shobara, Hiroshima, Japan

**Keywords:** 1-Lysophosphatidylcholine, Novozym® 435 (lipase B from *C. antarctica*), Ethanolysis

## Abstract

Efficient preparation methods for 1-lysophosphatidylcholine (1-LPC), a physiologically important compound, are lacking. Here, we established a method for 1-LPC preparation via Novozym® 435 (a lipase B from *Candida antarctica*)-catalyzed ethanolysis. Novozym® 435 showed *sn*-1 regiospecificity to phosphatidylcholine, although it does not exhibit regiospecificity to triacylglycerol. In particular, quantitative 1-LPC yields (96.5 ± 0.2 mol%) were reliably obtained in the presence of Novozym® 435 (100 wt% of PC), 97 % ethanol, in 72 h at 40 °C. During the reaction, acyl migration from 1-LPC to 2-LPC was rare. This novel synthetic method is expected to expand the practical applications of 1-LPC.

## Highlights

• 1-Lysophosphatidylcholine was prepared via Novozym® 435 (lipase B from *Candida antarctica*)-catalyzed ethanolysis.
• Novozym® 435 showed *sn*-1 regiospecificity to phosphatidylcholine.
• The water content of ethanol and high enzyme dose were key determinants of yields.
• The yield of 1-LPC at optimal reaction conditions was 96.5 ± 0.2 mol%.
• No acyl migration occurred during the reaction.

## 1 Introduction

Lysophosphatidylcholine (LPC) is a class of biomolecules derived from the cleavage of phosphatidylcholine (PC) via phospholipase A_1_ and A_2_ (PLA_2_) [1,2] or by the transfer of fatty acids to free cholesterol via lecithin-cholesterol acyltransferase [3]. LPC has two isomers, characterized by a hydroxyl group at the *sn*-1 position (1-LPC) or the *sn*-2 position (2-LPC). It exerts a variety of physiological functions; for example, it induces MCP-1 expression, increases inflammatory cytokines, disrupts mitochondrial integrity, activates macrophages, and induces oligodendrocyte demyelination [4,5]. However, to facilitate investigation regarding the physiological roles of these compounds and accelerate related research, a simple method for LPC synthesis is urgently needed.

To prepare 2-LPC, PLA_2_-catalyzed hydrolysis of PC is a simple and well-established method [6,7]. The *sn*-2 positional regiospecificity of PLA_2_ enables 2-LPC production in slightly alkali reaction conditions with calcium cations as co-activators of PLA_2_. This technique is often used to analyze the fatty acid composition at the *sn*-1, -2 positions of phospholipids [8]. The lipase-catalyzed acylation of glycerophosphocholine constitutes an alternative approach [[9], [10], [11]]. Carmen and Adlercreutz obtained 2-LPC with high conversion (>95 %) by acylation using several vinyl fatty acids as acyl donors and Novozym® 435 (lipase B from *Candida antarctica*) as a catalyst in a mixture of 50 % *t*-butanol and 0.5 mmol water at 25 °C for 100 h [9]. Mnasri et al. obtained 2-LPC with a yield of 75 % by acylation using oleic acid as an acyl donor and Lipozyme® RM-IM (*Rhizomucor miehei*) as a catalyst in a solvent-free system at 50 °C for 24 h (or 40 °C for 72 h) [11].

To prepare 1-LPC, phospholipase A_1_ (PLA_1_)-catalyzed hydrolysis has been applied by Lim et al. [12]. They obtained 83.7 mol% 1-LPC via PLA_1_ (*Thermomyces lanuginosus*)-catalyzed hydrolysis under a bi-phasic system of water and hexane [12]. However, the industrial application of PLA_1_ is limited by the high cost. In addition, quantitative yields have not been obtained using *sn*-1, 3 regiospecific lipase. 1-LPC production via lipase-catalyzed alcoholysis, first developed in 1994 by Sarney et al., has been demonstrated using Lipozyme® IM-60 (*Mucor miehei*) as a catalyst [13,14]. Quantitative yields (>98 %) of 1-LPC in several alcohols, such as ethanol, 2-propanol, and 1-butanol, have been obtained at 22 °C for 24 h [13]. In 2013, alcoholysis reactions catalyzed by PLA_1_ (*Thermomyces lanuginosus*), Novozym® 435, and Lipase PS (*Burkholderia cepacia*), were reported by Baeza-Jiménez et al. [15] with LPC yields of 50 %, 58.5 %, and 80 %, respectively. Although the LPC isomer obtained by Novozym® 435 was not reported in this previous study, it is expected to be 1-LPC because the enzyme shows *sn*-1 positional specificity to glycerophospholipids but no regiospecificity to triacylglycerols [9].

**Scheme 1:**
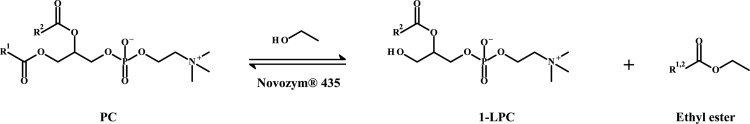
Preparation of 1-LPC via Novozym® 435 (lipase B from *Candida antarctica*)-catalyzed ethanolysis of PC (R; alkyl).

In this study, a simple preparation method of 1-LPC via Novozym® 435-catalyzed ethanolysis without any toxic solvents was demonstrated (scheme 1 ). The effects of the water content in ethanol, reaction temperature, and reaction time on 1-LPC yield were investigated.

## 2 Materials and methods

### 2.1 Materials

PC from soybean (Phospholipon 90 G) was purchased from H. Holstein Co., Ltd. (Tokyo, Japan). Novozym® 435 (lipase B from *Candida antarctica*) was purchased from MIK Pharm Co., Ltd. (Tokyo, Japan). LPC (l-α-lysophosphatidylcholine) from egg yolk, used as the 2-LPC standard, was obtained from FUJIFILM Wako Pure Chemical Corporation (Osaka, Japan).

All solvents and other chemicals used in this study were of analytical grade.

### 2.2 Enzymatic reaction

The typical enzymatic reaction was performed as follows. PC (50 μmol) was dissolved with 1 mL of 97 % ethanol, followed by the addition of Novozym® 435 (100 wt% of PC). The reaction was allowed to proceed at 40 °C and 900 rpm in the dark. After the reaction, the enzyme was removed by filtration. The reaction mixture was adjusted to 5 mL with ethanol containing 0.1 % formic acid to avoid acyl migration and subjected to high-performance liquid chromatography (HPLC) for calculation of the 1-LPC yield.

### 2.3 Calculation of the 1-LPC yield

The reaction mixture (Section 2.2) was subjected to HPLC to quantify 1-LPC, as described by Adlercreutz and Wehtje with slight modifications [16]. The HPLC system consisted of a Waters 2695 Separations Module (Milford, MA, USA) and a Refractive Index Detector Model 133 (GILSON, Middleton, WI, USA). An InertSustain® NH_2_ (4.6 × 250 mm, 5 μm; GL Sciences, Tokyo, Japan) column was used. Samples were then eluted by isocratic elution of the mobile phase at 95 % ethanol- and 20 mM oxalic acid with a ratio of 94:4 (v/v). The flow rate was maintained at 1.0 mL/min and the column temperature was maintained at 25 °C. Calibration curves were prepared with substrate PC and 2-LPC (*see* Section 2.1.) and levels of residual PC (mol%), 2-LPC (mol%), and 1-LPC yield (mol%, 2-LPC equivalent) were calculated. 1-LPC (mol%) was calculated by the Eq. (1). In these HPLC conditions, retention times (min) of PC, 1-LPC, and 2-LPC were 9.3, 11.5, and 12.6, respectively.

(1) 1-LPC (mol%) = 1-LPC (mol) / [PC (mol) +1-LPC (mol) + 2-LPC (mol)] x 100

### 2.4 Statistical analysis

All values are expressed as the means ± SD (n = 3). Statistical differences were determined using Scheffe’s tests, with a significance threshold of P < 0.05.

## 3 Results and discussion

### 3.1 Effect of ethanol concentration of the reaction solvent on 1-LPC yield

We first investigated the effect of the ethanol concentration on the ethanolysis reaction mediated by Novozym® 435. The reaction mixture included PC (50 μmol), solvents with different concentrations of ethanol (1.0 mL), and Novozym® 435 (100 wt% of PC). The reaction proceeded for 24 h at 60 °C and 900 rpm in the dark.

**Fig. 1:**
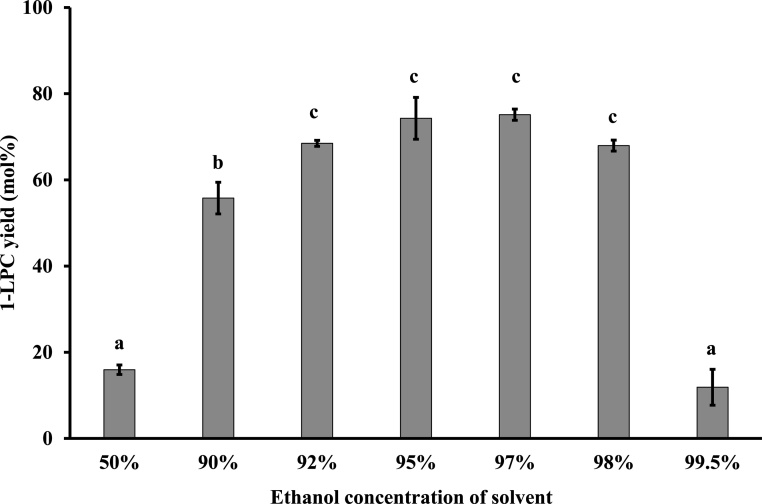
Effect of the ethanol concentration on 1-LPC yield. Reaction mixture; PC (50 μmol), solvent with different concentrations of ethanol (1.0 mL), and Novozym® 435 (100 wt% of PC). The reaction proceeded for 24 h at 60 °C, 900 rpm in the dark. Different letters indicate significant differences (P < 0.05).

The highest average yield (75.1 ± 1.3 mol%) was obtained using 97 % ethanol as a reaction solvent, although no significance differences in yield were observed for ethanol concentrations in the range of 92%–98% (Fig. 1). The yield decreased with the water content of the reaction solvent. In reaction solvents with high water contents, PC was not perfectly dissolved, resulting in low yields. For 50 % ethanol, only 16.0 ± 1.1 mol% 1-LPC was obtained and residual PC was 83.9 ± 0.9 mol%, suggesting that acyl migration and hydrolysis at the *sn*-2 position did not occur in this reaction system. At high concentrations of ethanol, the 1-LPC yield was also low; 11.2 ± 5.5 mol% 1-LPC was obtained in 99.5 % ethanol. This may be explained by enzyme denaturation caused by ethanol, which can absorb essential water [17], as small amounts of water are essential for the ethanolysis reaction to proceed using Novozym® 435 as a catalyst. These results are consistent with those of Sarney et al., who found that 5% water in ethanol was optimal although a high 1-LPC yield could be obtained up to 14 % [13].

Based on these results, 97 % ethanol was selected as the reaction solvent in the reaction system.

### 3.2 Effect of reaction temperature on 1-LPC yield

An ethanolysis reaction was performed under different temperatures. The reaction mixture consisted of PC (50 μmol), 97 % ethanol (1.0 mL), and Novozym® 435 (100 wt% of PC). The reaction proceeded for 24 h at 4–60 °C at 900 rpm in the dark.

**Fig. 2:**
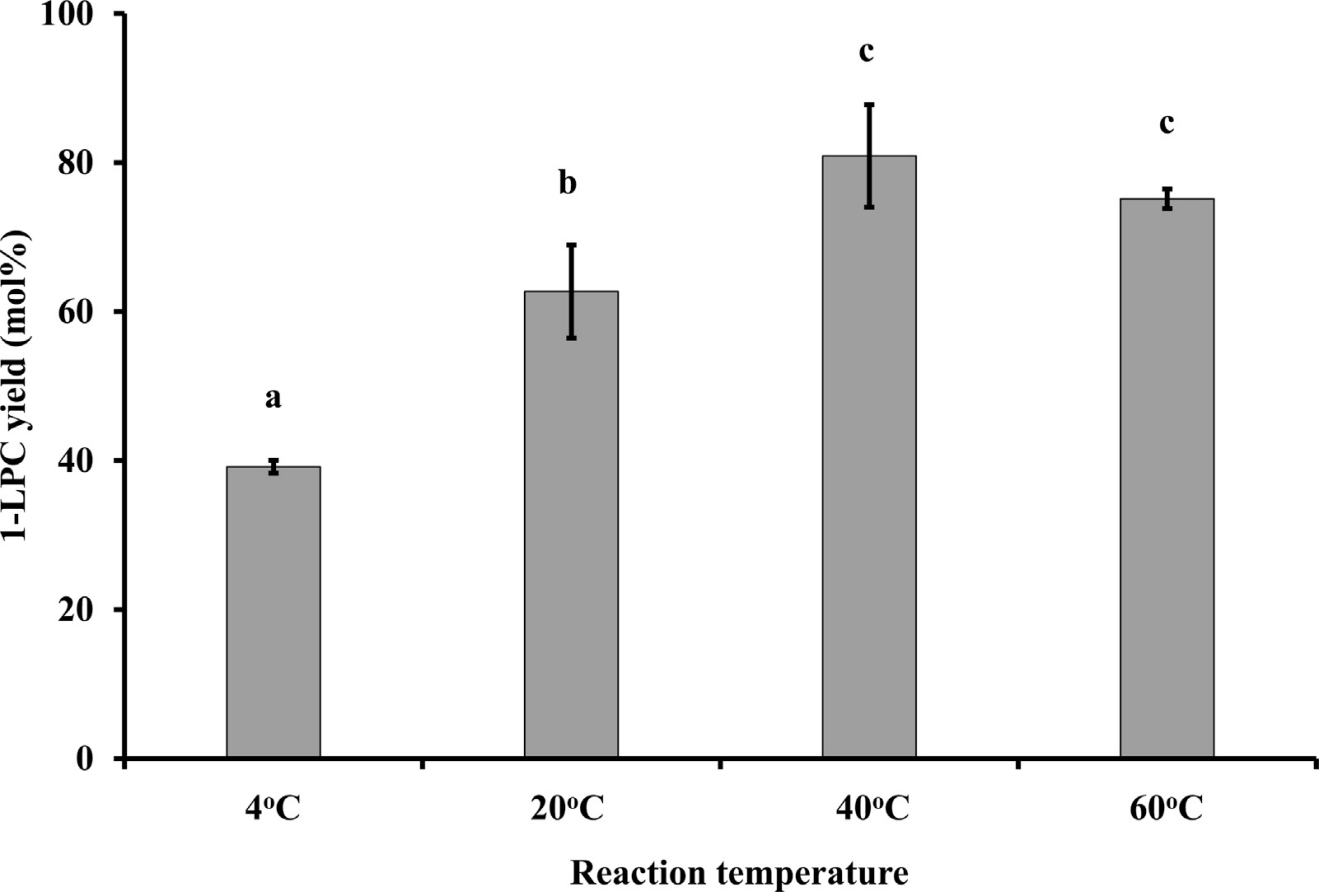
Effect of the reaction temperature on 1-LPC yield. Reaction mixture; PC (50 μmol), 97 % ethanol (1.0 mL), and Novozym® 435 (100 wt% of PC). The reaction proceeded for 24 h at various temperatures and 900 rpm in the dark. Different letters indicate significant differences (P < 0.05).

The highest 1-LPC yields were obtained at 40 °C (80.9 ± 8.7 mol%), although the yields at this temperature did not differ significantly from those at 60 °C (Fig. 2). At 20 °C, the 1-LPC yield was 62.7 ± 8.5 mol% which was moderate, and at 4 °C, the yield was lower. These results showed that the reaction system is suitable for a substrate containing unsaturated fatty acids, such as marine phospholipids with eicosapentaenoic acid and docosahexaenoic acid, which are sensitive to oxidative deterioration.

Baeza-Jiménez et al. also investigated the effect of temperature on the Novozym® 435-catalyzed ethanolysis of PC, showing that the degree of ethanolysis increased clearly as the reaction temperature increased from 40 °C to 60 °C [15]. They used Novozym® 435 with 15 wt% of PC, compared with 100 wt% of PC in our study. This difference in enzyme dose might influence reaction temperature because the reaction rate is high in reaction systems with high enzyme doses. Nevertheless, it is challenging to precisely clarify the effect of temperature.

The 1-LPC yield did not differ significantly between reaction temperatures of 40 °C and 60 °C; accordingly, 40 °C was selected as the optimal reaction temperature in this system to avoid acyl migration at high temperatures.

### 3.3 Effect of enzyme dose on 1-LPC yield and residual PC

Next, the effect of enzyme dose on 1-LPC yield and residual PC was investigated. The reaction mixture consisted of PC (50 μmol), 97 % ethanol (1.0 mL), and different doses of Novozym® 435 (12.5–100 wt% of PC). The reaction proceeded for 24 h at 40 °C, 900 rpm in the dark.

**Fig. 3:**
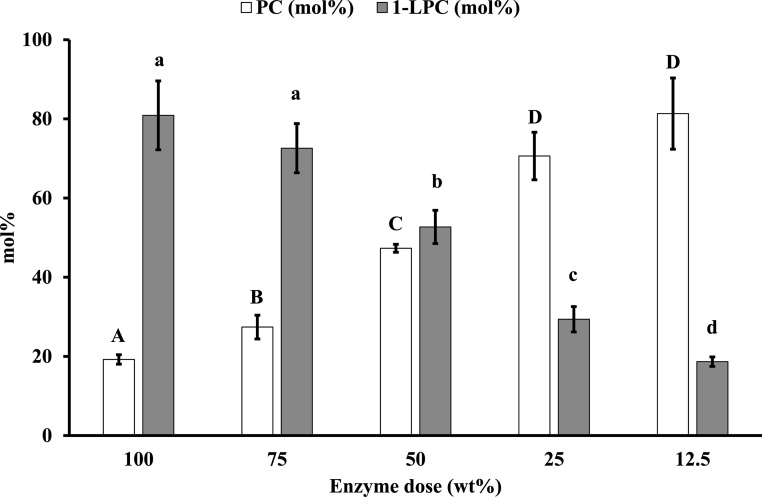
Effect of enzyme dose on 1-LPC yield and residual PC. Reaction mixture; PC (50 μmol), 97 % ethanol (1.0 mL), and Novozym® 435 (12.5–100 wt% of PC). The reaction proceeded for 24 h at various temperatures and 900 rpm in the dark. Different letters indicate significant differences (P < 0.05).

1-LPC yield decreased with decreasing enzyme dose (Fig. 3). In accordance with decreasing 1-LPC yield, residual PC increased whereas no 2-LPC yield was observed in any trial. The highest 1-LPC yield was obtained when 100 wt% of Novozym®435 was used (this value was obtained from the trial shown in Fig. 2). Notably, even the use of 50 wt% of Novozym® 435 afforded significantly lower 1-LPC yield than that obtained with 100 or 75 wt%. When 12.5 wt% of Novozym® 435 was used, the standard amount applied in current Novozym® 435-mediated processes, < 20 mol% of 1-LPC yield was obtained. Yang et al. reported 97.7 % yield of 1-LPC via Novozym® 435 mediated ethanolysis of PC using only 10 wt% of biocatalyst loading [18]. However, we have not been able reproduce this result.

This result indicated that this reaction system requires a substantial enzyme dose to reliably obtain high 1-LPC yield, likely because short chain alcohols, such as methanol and ethanol, tend to dehydrate enzyme protein and thereby deactivate the enzyme [19]. Although immobilized enzymes such as Novozym® 435, which is immobilized on acrylic resin, can overcome this problem by maintaining their active structure [20], a large enzyme dose was required in a reaction media containing high concentration of ethanol which is essential to shift reaction equilibrium to produce 1-LPC.

### 3.4 Effect of reaction time on 1-LPC yield

Finally, the effect of reaction time was investigated using a mixture consisting of PC (50 μmol), 97 % ethanol (1.0 mL), and Novozym® 435 (100 wt% of PC). The reaction was continued for 72 h at 40 °C, 900 rpm in the dark.

**Fig. 4:**
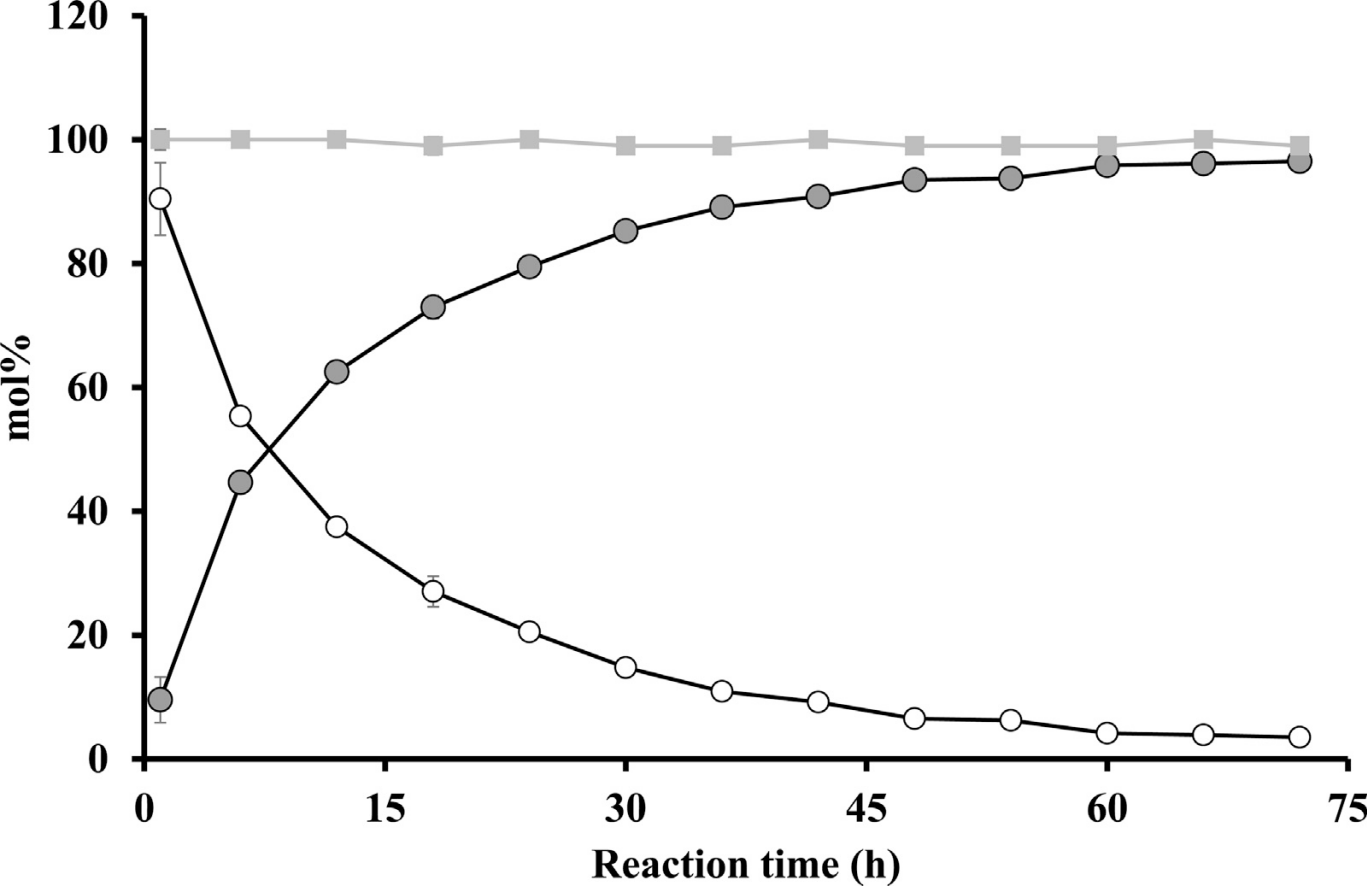
Time course of Novozym® 435-catalyzed ethanolysis of PC. Reaction mixture; PC (50 μmol), 97 % ethanol (1.0 mL), and Novozym® 435 (100 wt% of PC). The reaction proceeded for 1–72 h at 40 °C, 900 rpm in the dark. Symbol: 1-LPC (mol%), open circle; PC (mol%), closed circle; 1-LPC + PC (mol%), square.

1-LPC yield increased as the reaction time increased and reached a plateau at 60 h (Fig. 4). The maximum 1-LPC yield of 96.5 ± 0.2 mol% was obtained at 72 h. Residual PC substrate decreased as the reaction time increased. The total amount of 1-LPC + residual PC was nearly 100 mol% during the reaction, suggesting that acyl migration from 1-LPC to 2-LPC was rare in this reaction system.

Alternatively, Baeza-Jiménez et al. reported that the degree of ethanolysis increases until 48 h and apparently decreases thereafter at several reaction temperatures (30–60 °C) [15]. We note that acyl migration from 1-LPC to 2-LPC might occur in this previously established reaction system. In addition, the discrepancy between the results of the previous [15] and current study may arise from the differences in the concentration of the reaction system. Specifically, Baeza-Jiménez et al. used an enzyme concentration of 15 wt% (to PC) in 5 mL of 95 % ethanol (150 mg PC /mL), whereas we applied 100 wt% enzyme in 1.0 mL of 97 % ethanol (approximately 40 mg PC /mL). However, additional research is required to clarify this issue.

## 4 Conclusion

1-LPC was successfully prepared under the following optimized reaction conditions: PC (50 μmol), 97 % ethanol (1.0 mL), and Novozym® 435 (100 wt% of PC), with a 72-h reaction time at 40 °C and 900 rpm in the dark. This resulted in a 1-LPC yield of 96.5 ± 0.2 mol%. In this reaction system, Novozym® 435 showed regiospecificity at the *sn*-1 position of PC and acyl migration from 1-LPC to 2-LPC was rare.

## CRediT authorship contribution statement

**Sayumi Yasuda:** Investigation. **Yukihiro Yamamoto:** Conceptualization, Methodology, Writing - original draft.

## Declaration of Competing Interest

None.
